# Complement Inhibitor Therapy for Myasthenia Gravis

**DOI:** 10.3389/fimmu.2020.00917

**Published:** 2020-06-03

**Authors:** Khaled Albazli, Henry J. Kaminski, James F. Howard

**Affiliations:** ^1^Department of Neurology, George Washington University, Washington, DC, United States; ^2^Department of Neurology, University of North Carolina at Chapel Hill, Chapel Hill, NC, United States

**Keywords:** complement, C5, myasthenia (myasthenia gravis—MG), eculizumab, zilucoplan

## Abstract

Complement activation as a driver of pathology in myasthenia gravis (MG) has been appreciated for decades. The terminal complement component [membrane attack complex (MAC)] is found at the neuromuscular junctions of patients with MG. Animals with experimental autoimmune MG are dependent predominantly on an active complement system to develop weakness. Mice deficient in intrinsic complement regulatory proteins demonstrate a significant increase in the destruction of the neuromuscular junction. As subtypes of MG have been better defined, it has been appreciated that acetylcholine receptor antibody-positive disease is driven by complement activation. Preclinical assessments have confirmed that complement inhibition would be a viable therapeutic approach. Eculizumab, an antibody directed toward the C5 component of complement, was demonstrated to be effective in a Phase 3 trial with subsequent approval by the Federal Drug Administration of the United States and other worldwide regulatory agencies for its use in acetylcholine receptor antibody-positive MG. Second- and third-generation complement inhibitors are in development and approaching pivotal efficacy evaluations. This review will summarize the history and present the state of knowledge of this new therapeutic modality.

## Introduction

Myasthenia gravis (MG) is an autoimmune disease in which the postsynaptic membrane is depleted of acetylcholine receptor (AChR) causing a compromise of neuromuscular transmission (1). Antibodies directed against the AChR are the primary driver of pathology in most patients. In those patients without detectable circulating AChR antibodies, the muscle-specific kinase (MuSK) and low-density lipoprotein receptor-related protein 4 (LRP4) have been identified as pathological targets, and other neuromuscular junction proteins are under investigation (2, 3).

**Figure 1:**
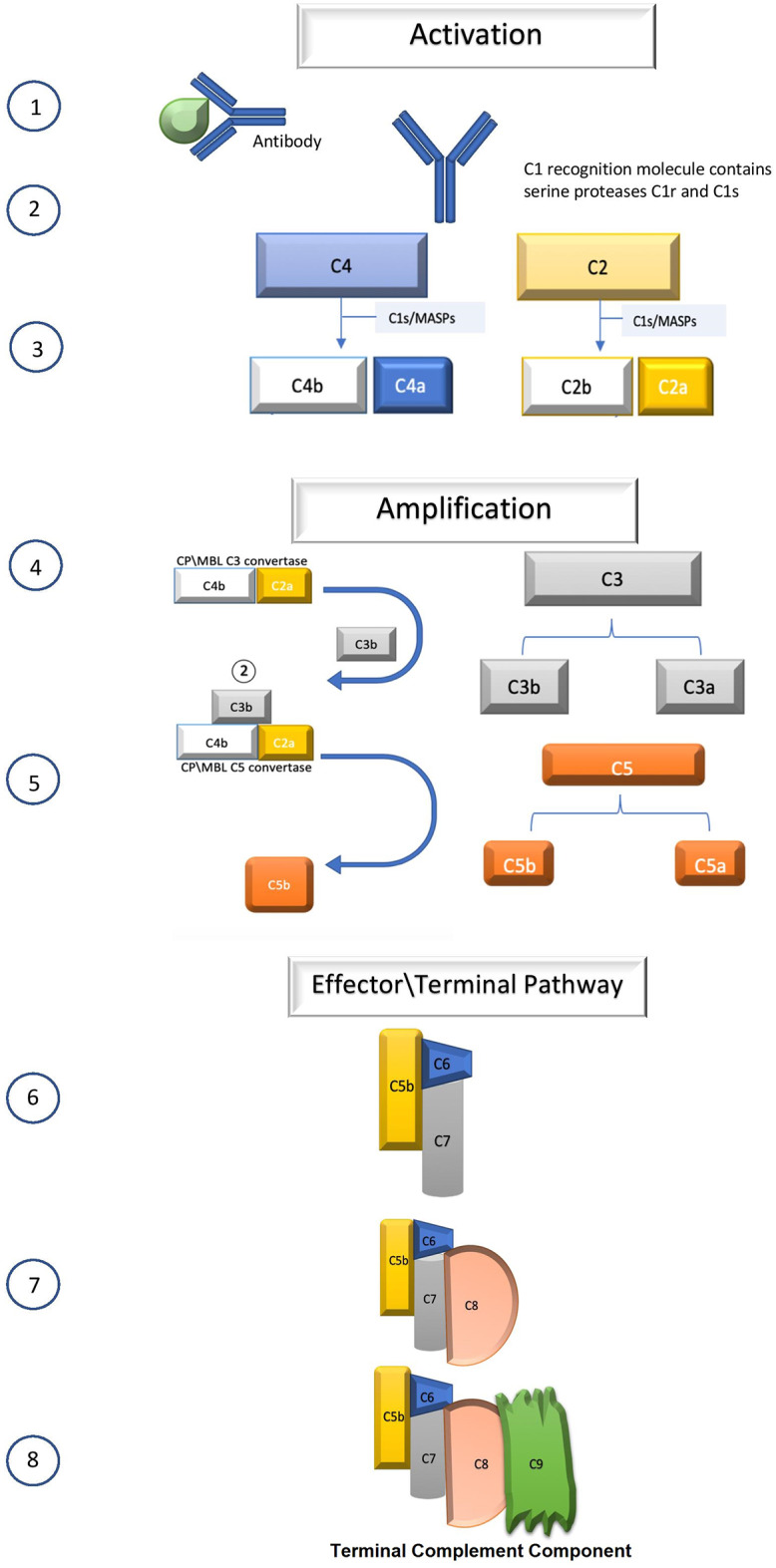
Schematic of complement cascade (see text for details).

Myasthenic autoantibodies are polyclonal with variations in subclasses, epitope targets, binding avidity, and pathogenic mechanisms (4). The characteristics of the population of autoantibodies among individual patients vary and change over the course of the disease. The mechanisms of pathology are best understood for MuSK and AChR antibodies. The predominant subclass of MuSK autoantibodies is immunoglobulin G (IgG)4, which lacks the ability to activate the complement cascade and is considered to be functionally monovalent. MuSK is a receptor tyrosine kinase crucial for formation and maintenance of neuromuscular junction, and MuSK autoantibodies interfere with clustering of the AChR. Studies starting in the 1970s demonstrated the three pathogenic mechanisms for AChR antibodies: blockade of AChR channel function, cross-linking of AChR by the divalent AChR antibody (antigenic modulation), and complement activation (5–8). Antibody binding to a variety of determinants of the multimeric AChR may result in a functional block of AChR channel function or prevent acetylcholine binding. Interestingly, most of the antibodies do not directly block the transmitter binding site on AChR. In antigenic modulation of AChR, binding of antibody and subsequent cross-linking lead to an increase in the natural degradation cycle of the receptors. The third, and likely most critical mechanism, is for AChR antibodies to activate complement with ultimate formation of the terminal complement component (TCC) causing damage to the muscle membrane (Figure 1) (3, 9). The role of complement activation in patients without AChR antibodies is poorly defined (10).

This review will provide a broad overview of the complement system, the preclinical data that support the role of complement in driving MG pathology, and application of complement inhibitors in the treatment of MG.

## The Complement Cascade

**Figure 2:**
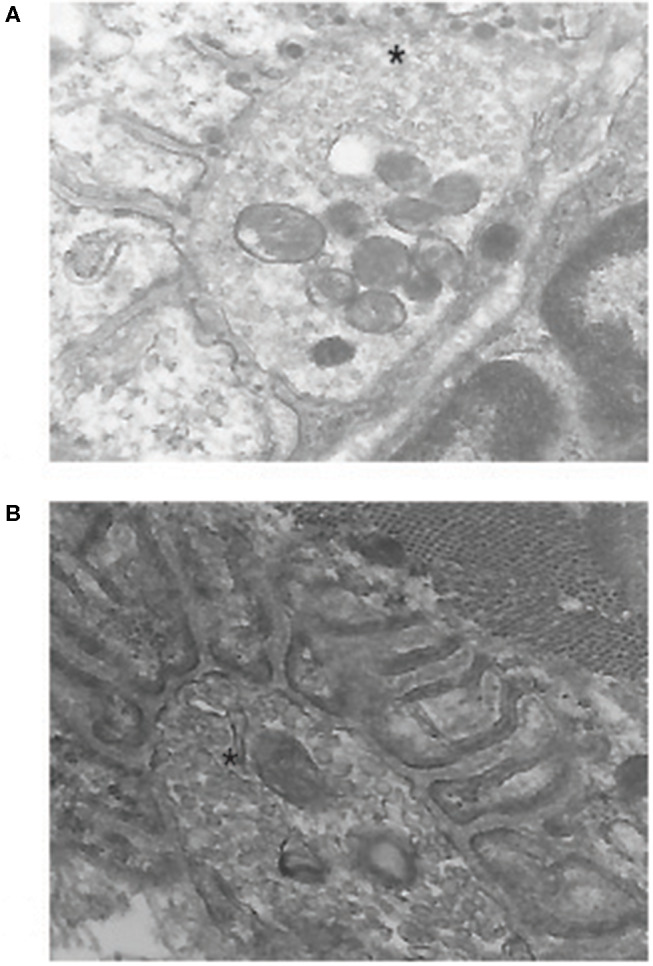
Electron micrographs of mouse neuromuscular junction. **(A)** shows a junction from an experimental autoimmune myasthenia gravis (EAMG) animal. Note the electron-dense material in the synaptic cleft and the loss of regular postsynaptic folds that are seen in a normal junction in **(B)**. The* marks the nerve terminal. Previously unpublished images by one of the authors (HK).

Complement is part of the innate immune system and a key mediator of antibody function through the ultimate formation of the TCC, which serves to rupture bacterial and cellular membranes as well as signaling phagocytic cells to remove pathogens (11, 12). Over 30 proteins compose the complement cascade (Figure 1), which is activated by either antibody (classical pathway), spontaneously formed C3b (alternative), and binding of lectins found on bacterial cell surfaces (alternative). In human AChR Ab-positive MG, the classical pathway is initiated (activation step) when IgG1 or IgG3 (less so IgG2) autoantibodies attached to the AChR bind C1q. C1q binds the Fc domain of the antibody, leading to the autoactivation of C1r and the subsequent activation of C1s. C1s then cleaves C4 to C4a and the larger C4b. The C1s and C1r combine with C4b to form C14b. The amplification phase occurs when C14B enzymatically converts C2 to C2a and C2b. The C14B combines C2a to form C14b2a, which is also known as C3 convertase. Spontaneous hydrolysis of C3 may also occur, and the formation C3b combining with Factor B produces C3 convertase of the alternative pathway (Figure 1). C3 convertase enzymatically cleaves C3 into C3a and C3b. C3b with the C3 convertase forms C14b2a3b, which is the C5 convertase. The C5 convertase then cleaves C5 to C5a and C5b. The C5b combines with C6, C7, C8, and C9 to form C5b6789, which is the effector mechanism of the complement system. TCC formation produces focal lysis of the neuromuscular junction with loss of AChR and postsynaptic folds (Figure 2) (10).

The complement cascade may be spontaneously activated with potential devastating cell injury, which explains that need for inhibitory regulators that are found on nearly all cell surfaces (13). The decay accelerating protein [decay accelerating factor (DAF), CD55] and CD59 are the primary cell surface inhibitors in humans and can be found localized to the neuromuscular junction (14, 15). DAF is a membrane-bound protein that dissociates C3 and C5 convertases, while CD59 interferes with TCC formation. Interestingly, complement regulators are expressed at lower levels in extraocular muscle and could account for the differential involvement of these muscles by MG (16, 17).

## Evidence of Complement as an Effector Mechanism in Myasthenia Gravis and Its Animal Models

Several lines of evidence support activation of the complement system is critical to the pathology of human MG and animal models of MG. An important early observation was the identification of C3 and C9 localized to fragments of degenerated junction folds where identified in MG patient neuromuscular junctions (Figure 2) (8, 18–20). Depletion of serum complement components, C3 and C4, is observed in patients, terminal components of complement are present in MG patient sera, and sera induce complement-mediated lysis of cultured myotubes (21–23). The effectiveness of C5 inhibition in human trials, which will be discussed later in this review, offers further compelling evidence that complement is a critical mediator of MG.

The relative contribution of non-complement-mediated mechanisms to human MG is poorly defined. Administration into mice of even large quantities of human autoantibodies (equal to 50% of the total mouse circulating mouse IgG) produces only mild weakness (7, 24). Because human complement is not co-administered, weakness would likely only develop from antigenic modulation or impairment of AChR function. Miniature endplate potentials are not altered by infusion of human MG serum, when rats are rendered intolerant to human immunoglobulin (IG) (25). Studies using rodents are influenced by the ability of human AChR antibody to bind rodent AChR, leading to the potential to underestimate the effect of AChR blockade or antigenic modulation.

Two basic animal models are used to study MG. Passive transfer MG (PTMG) consists of administration of autoantibodies (9, 26). For AChR-antibody PTMG, monoclonal antibodies, syngeneic polyclonal serum, and highly concentrated human MG sera with a source of active complement have been used. Within 24–72 h of receiving antibody, rodents develop mild to severe weakness depending on species and antibody properties as well as having complement components deposited at the neuromuscular junction. A significant deficiency of PTMG is that it only interrogates the final antibody effector mechanism and not the loss of tolerance that leads to antibody production. Experimental autoimmune MG (EAMG) involves immunization with purified AChR or peptide fragments of the AChR. Over weeks to months depending on species, an antibody response develops directed toward the administered AChR and then toward the native protein. Complement components with antibody are then found at the neuromuscular junction, and animals develop weakness with neuromuscular transmission deficits consistent with a myasthenic phenotype.

Studies of complement depletion were the first approaches to assess complement mechanisms in the pathology of EAMG. Early experiments demonstrated that cobra venom injected, either during the acute phase of the active EAMG in rats or prior to AChR antibody administration in PTMG, leads to a reduction of disease severity (7, 27). C6-deficient rats do not demonstrate weakness or form TCC on the postsynaptic membrane in response to AChR antibody administration, while exogenous C6 restores TCC assembly and weakness (28). Engineered knockout of C3 and C4 in mice leads to a reduction of weakness from EAMG and preserved AChR density at the neuromuscular junctions (29). Interestingly, the animals show some reduction of complement fixing antibody subclasses, which suggests that the complement system influences cellular autoimmunity (30). In contrast, C5 knockout mice develop comparable levels of circulating AChR-specific antibodies, but C5 knockout mice have no weakness or junctional injury (31). Taken together, the data suggest that activation of complement is the overwhelming driver of EAMG pathology since weakness is not evident when complement activity is ablated. This is likely not to be the case in humans. In contrast to the preservation of function with removal of complement components, the engineered ablation of cell surface complement regulatory proteins leads to severe pathology when PTMG is induced (32–34).

## Preclinical Validation for Complement Inhibitor Therapy in Myasthenia Gravis

Exogenous provision of complement inhibitors as a potential therapy has a long history in MG. In 1989, a monoclonal antibody to C6 was administered to PTMG rats and reduced weakness, preserved body weight, and preserved normal electrophysiological properties (35). A soluble complement receptor 1 (sCR1) to Lewis rats at the time of PTMG induction found a reduction of weakness and retained AChR density (36). Anti-C5 antibody treatment was found to limit PTMG severity (37), which justified application of C5-focused treatments in humans (see below). Another C5 inhibitor is coversin (rEV576), which is a recombinant protein derived from ticks and effective in moderating disease severity of PTMG and EAMG (38). Coversin has moved to human clinical trials for paroxysmal nocturnal hemoglobinuria (PNH) but is not being developed for MG. Targeting complement inhibition to the site of pathology, the neuromuscular junction, reduces PTMG severity and has the advantage of limiting systemic complement inhibition (39, 40).

In addition to antibody-based targeting of complement components, siRNA therapies have been used to suppress C component expression. Reduction of the C2 component of complement reduced serum complement activity in mice with a resultant reduction of weakness, retention of AChR, and reduced TCC deposition in EAMG mice (41). Targeting of C5 expression by the liver leads to similar findings in rat EAMG (42). Thus, far, these approaches have not moved into human assessment.

## Human Studies of Complement Inhibition

Patients with generalized MG (gMG) have a large disease burden with an increased risk of disease exacerbation, hospitalization, intensive care stay, and intolerable side effects to the medications used in their treatment (43–45). In addition, 10–15% of patients with AChR antibody–positive (AChR+) gMG are refractory to the most common immunotherapeutic paradigms (46, 47). As such, there is a need for target-specific therapies with improved adverse event profiles. Progress has been made over the last 12 years with the development of new novel therapeutics that attempt to address these issues: the inhibition of complement targets and the neonatal Fc receptor (FcRn).

## Clinical Trials

To date, there have been three completed clinical trials of complement inhibition in gMG: two phase 2 trials and one phase 3 trial. All have targeted C5 with the goal of blocking terminal complement activation, preventing the pro-inflammatory effects of C5a and C5b and the subsequent formation of the terminal complement component or membrane attack complex (C5b-9) (28). Each of these trials focused on AChR+ generalized MG as the predominant antibody subclass, IgG3, is a potent activator of complement.

### Phase 2 Trials

**Figure 3:**
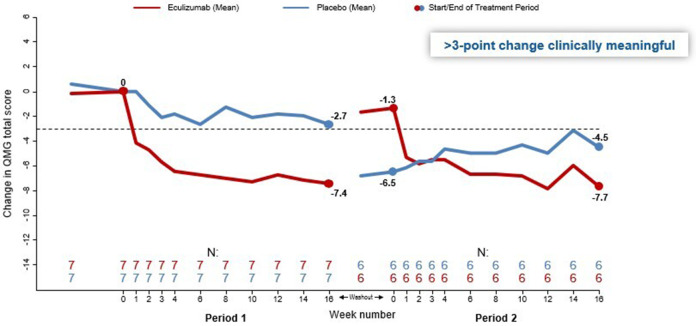
Quantitative myasthenia gravis (QMG) score change from baseline over 12 weeks for eculizumab vs. placebo. Using patient data at the end of both period visits, overall change in mean QMG total score was significantly different between eculizumab and placebo (−7.92 vs. −3.67; paired t-test *p* = 0.0144). Using patient data at all visits, overall change in mean QMG total score was significantly different between eculizumab and placebo (−6.43 vs. −3.18; repeated-measures mixed model *p* < 0.0001). Modified from Howard et al. (48).

The initial phase 2 trial (NCT00727194), sponsored by Alexion Pharmaceuticals, was a prospective, double-blind, placebo-controlled crossover design of 14 AChR+, gMG treatment-refractory patients [Myasthenia Gravis Foundation of America (MGFA), Classes II–IVa)] initially treated for 16 weeks (Period 1) followed by a 5-week washout period and then crossed over (Period 2) to the other investigational product for an additional 16 weeks (48). Patients were required to have persistent weakness despite treatment with at least two immunosuppressive drugs for at least 1 year and could not have received intravenous immunoglobulin (IVIg), plasma exchange, rituximab, or thymectomy within 2, 3, 6, or 12 months of screening, respectively. This study used the full sized, humanized monoclonal antibody eculizumab that specifically binds to and inhibits cleavage of C5 into C5a and C5b (49). Standard of care was maintained through the duration of the study. Study subjects received either eculizumab 600 mg or a matching placebo infused IV for 4 consecutive weeks, followed by the administration of 900 mg IV of eculizumab or matching placebo every 2 weeks. Six of seven patients (86%), treated for 16 weeks, met the primary efficacy endpoint of a 3-point reduction in the Quantitative MG (QMG) score vs. 50% of placebo-treated patients. Four of seven (57%) of patients treated with eculizumab had an 8-point improvement in total QMG score compared to only one of seven (14%) who received placebo. Of note, eculizumab-treated patients did not return to their baseline QMG scores despite a 5-week washout prior to beginning Period 2 (Figure 3). This suggests a carryover effect, although the mechanism of such is not known.

**Figure 4:**
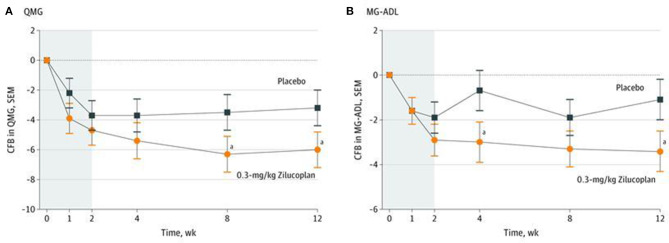
Change from baseline over 12 weeks for 0.3 mg/kg zilucoplan vs. placebo. **(A)** Change from baseline to week 12 in Quantitative Myasthenia Gravis (QMG) Score. **(B)** Change from baseline to week 12 in MG Activities of Daily Living (MG-ADL) Score. Modified from Howard et al. (50). *p < 0.10.

The second phase 2 trial (ClinicalTrials.gov Identifier: NCT03315130), sponsored by Ra Pharmaceuticals was a prospective, double–blind, placebo-controlled study of 44 AChR+ gMG patients over 12 weeks followed by an open-label extension (OLE) trial that continues at this time (50). This study used zilucoplan, a small (3.5-kDa), 15-amino acid macrocyclic peptide, that binds to C5 with high affinity and specificity and also binds to the domain of C5 that corresponds to C5b and thereby also blocks binding of C5b to complement component C6 (51). Patients were randomized 1:1:1 to zilucoplan 0.1 mg/kg, zilucoplan 0.3 mg/kg, or matching placebo self-administered subcutaneously daily for 12 weeks, and eligible participants could enter the OLE. Entry criteria were like the Alexion phase 2 trial in age, disease severity, and baseline QMG scores, but there was no requirement to be treatment refractory. Standard of care was maintained throughout the study. Rapid, robust, and a sustained response was seen in the zilucoplan-treated group. The primary efficacy measure was the change in QMG score from baseline to week 12; a 6-point change in the 0.3-mg/kg zilucoplan group compared with −3.2 points in the placebo-treated group (*p* = 0.05). Onset of improvement was as early as 1 week (Figure 4). The 0.1-mg/kg zilucoplan dose demonstrated a slower onset of action and a less pronounced effect when compared to the higher zilucoplan dose although still a clinically meaningful response when compared to placebo. Similar findings were seen when comparing the change in MG Activities of Daily Living (MG-ADL) score from baseline to week 12 in both arms compared to placebo.

### Phase 3 Trials

REGAIN (NCT01997229), a phase 3 trial with an OLE (NCT02301624) also used the monoclonal antibody eculizumab (52, 53). This prospective, double–blind, placebo-controlled study enrolled 125 treatment-refractory AChR+ gMG patients of moderate to severe severity (MGFA Classes II–IV) at 72 centers in Asia, Europe, Latin America, and North America. Treatment refractory was defined as having persistent weakness despite treatment with at least two immunosuppressive therapies (ISTs) or one IST with the requirement of chronic plasma exchange or IVIg. Subjects were randomized 1:1 to either eculizumab or a matched control for 26 weeks. Eculizumab was administered IV; an induction dose of 900 mg weekly for four doses (day 1, weeks 1–3) and a maintenance dose of 1,200 mg every other week beginning on week 4. Subjects who completed the 26-week REGAIN study were eligible to participate in the OLE, and 117 patients elected to do so (53).

The primary efficacy endpoint was the change in the MG-ADL score from baseline to week 26 for eculizumab treated subjects compared to placebo measured by worst-rank analysis of covariance (ANCOVA) analysis. Multiple prespecified secondary endpoints included the change in QMG total score from baseline, responder analysis of the MG-ADL and QMG scores for those with at least a 3-point and 5-point improvement, respectively, and changes in the MG Composite (MGC) and MG Quality of Life 15 (MG-QoL15) scores from baseline.

**Figure 5:**
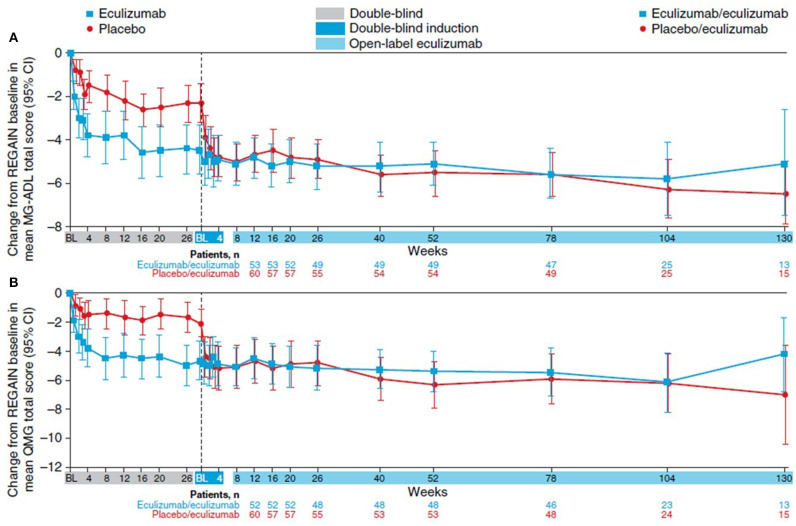
REGAIN and open-label extension (OLE). **(A)** The change in MG-ADL score from REGAIN baseline to OLE week 130. **(B)** The change in QMG score from REGAIN baseline to OLE week 130. BL, baseline; CI, confidence interval; MG-ADL, Myasthenia Gravis Activities of Daily Living; QMG, Quantitative Myasthenia Gravis Score. Vertical dash line represents the transition from REGAIN to the OLE. Adapted from Muppidi et al. (53).

The primary endpoint, the mean ranked difference in the change in MG-ADL score between baseline and placebo at week 26 was not significant despite significant change in 18 of 21 secondary measures (Table 1). Rapid, robust, and durable improvement was seen in the MG-ADL of eculizumab-treated patients compared to placebo (Figure 5). Improvement was noted during the week following their first infusion, was maximal around 12 weeks, and remained durable for the duration of the 130-week observation. A similar profile was seen with the QMG score (Figure 5), MGC, and MG-QoL15, although the latter has a slightly slower time course (data not shown). During the trial, 56% of patients achieved the clinical state of minimal manifestations. Additionally, exacerbation rates were reduced by 75% (*p* = 0.0001) from the year prior to study entry. Patients who received placebo during the REGAIN trial had a similar response when transitioned to eculizumab in the OLE. The speed and degree of improvement mimicked those seen in the REGAIN trial.

**Table 1:** REGAIN totality and consistency of analyses.

	**Primary/secondary endpoints**		**Sensitivity analyses**		
**Outcome measure**	**Worst-Rank ANCOVA**	**Responder analysis**	**Repeated measures at week 26 (IST as covariate**	**Change from baseline at week 26 or LOCF ANCOVA**	**Wost-rank ANCOVA sensitivity**
MG-ADL	0.0698*	0.0229	0.0058 (0.0077)	0.0390	0.0800*
QMG	0.0129	0.0018	0.0006 (0.0007)	0.0032	0.0169
MGC	0.1026*	N/A	0.0134 (0.0168)	0.0406	0.1084*
MG-QOL15	0.0281	N/A	0.0010 (0.0009)	0.0152	0.0328

REGAIN Study results of 22 prespecified measures demonstrating the totality of the data supporting the positive effects of complement inhibition in the treatment of myasthenia gravis (MG). Analyses marked with an* reflect the bias imposed by the worst-rank analysis of covariance (ANCOVA) methodology. Adapted from Howard et al. (52).

Currently, multinational phase 3 trials are underway with zilucoplan (NCT04115293) administered daily subcutaneously and ravulizumab (NCT03920293), a monoclonal antibody developed by Alexion Pharmaceuticals, administered IV every 8 weeks. The primary efficacy endpoint will be the change from baseline in the MG-ADL score at 12 and 26 weeks, respectively.

### *Post hoc* Analyses

Numerous *post hoc* analyses have been performed on the REGAIN dataset. Eculizumab demonstrated rapid and significant improvement in ocular, bulbar, respiratory, and limb domains at week 26 compared to baseline and through 130 weeks of the OLE phase of the study. The improvements were observed at week 26 for both ocular and gross motor, by week 20 bulbar domains, and by week 12 for the respiratory domain and remain stable through 130 weeks (54).

The ability to reduce IST use during the OLE phase of the study was noted. At baseline, 115 of 117 (98.3%) study subjects were using at least one immunosuppressive drug (55). This was reduced to 103/117 (88%) at the time of their last assessment. Additionally, significant reductions in the total daily dose of corticosteroids, azathioprine, and mycophenolate mofetil were demonstrated.

The REGAIN study demonstrated that eculizumab-treated patients had significantly more improvement in the extended Neuro-QOL Fatigue scale, a patient-reported assessment of fatigue, when compared to the placebo arm. Eculizumab-treated patients had a 16.3-point change from baseline compared to a 7.7-point change in the placebo group at week 26 (56). This improvement was sustained through week 52 (data cut) of the open-label study. Strong correlations were observed between the Neuro-QOL Fatigue scale and the MG-ADL, MG-QoL15, and QMG scores.

Subgroup analysis of the REGAIN population previously dependent upon IVIg demonstrated rapid and robust improvement in their MG outcome measures with fewer disease exacerbations when treated with eculizumab in both the blinded and open-label portions of the study (57). Treatment with eculizumab was associated with a 65% reduction (*p* = 0.0057) in MG exacerbation rates during the REGAIN trial compared to the placebo arm (58). Further, there was a 66% reduction in hospitalization rates (*p* = 0.0316) as well as the need for rescue therapy (*p* = 0.0072).

The MGFA post intervention status of minimal manifestations (MMs), the presence of minimal nonfunctional weakness or no weakness on clinical examination, is the stated goal of several treatment guidelines (59–61). The REGAIN study demonstrated that at week 26, 25% of eculizumab-treated patients achieved a state of MM vs. 13.3% of those treated with placebo. A total of 57.1% of patients achieved this status through 130 weeks of OLE.

Current definitions of minimal symptoms rely on the physical examination and not that of the patient's impression of their disease. The concept of Minimal Symptom Expression (MSE) has been developed as a more meaningful way to assess patient response without the need for clinical examination. MSE is defined as an MG-ADL score of 0 or 1. Vissing et al. (62) have reported more patients receiving eculizumab than placebo met the criteria of MSE (21.4 vs. 1.7%, *p* = 0.0069) at week 26 of treatment during the REGAIN trial. This was maintained through 130 weeks of the OLE, and a similar number of patients in the placebo/eculizumab and the eculizumab/eculizumab groups achieved “minimal symptom expression” (MG-ADL: 22.9 and 27.8%, respectively, *p* = 0.7861) at week 130 of treatment. These findings suggest that MSE may be a potential endpoint for future clinical trials or in the clinic.

By week 12 in the REGAIN study, 67.3% of eculizumab-treated patients had at least a 3-point change in the MG-ADL score, and 56.1% had at least a 5-point change in the QMG score. At the end of OLE, 84.7% of eculizumab-treated patients reached the MG-ADL criteria, and 71.4% of patients reached the QMG criteria. Then, 15.3% and 28.6% of patients did not achieve MG-ADL or QMG response, respectively. These data would suggest that there is a subpopulation of patients whose response is much slower (63).

## Safety

The inhibition of C5 increases the risk of *Neisseria* infection. For this reason, patients treated with anti-C5 inhibitors must be vaccinated against *Neisseria* meningitides with both the quadrivalent and B-serotype vaccines according to the guidelines published by the CDC Advisory Committee on Immunization Practices. Information can be obtained from the Centers for Disease Control at https://www.cdc.gov/meningococcal/about/soliris-patients.html. Patients must be educated on the presenting symptoms of meningococcal meningitis, and all should carry an informational safety card to present at each health encounter.

Patients who are unable to be vaccinated at least 14 days prior to their initial dose of a C5 inhibitor must be treated with antibiotics. Treatment with C5 inhibitors should not be delayed during this period. Care must be taken to avoid the fluoroquinolone and macrolide classes as these have the potential to acutely worsen myasthenic weakness. The role of long-term prophylactic antibiotic therapy for MG patients treated with a complement inhibitor remains controversial. Pediatric myasthenic populations are not approved currently for the use of C5 inhibitors. Should this approval be forthcoming, additional vaccinations against *Streptococcus pneumoniae* and *Haemophilus influenzae* type b are recommended.

In all three trials, the safety profile for the three C5 inhibitors was like those seen in PNH and atypical hemolytic uremic syndrome (aHUS), most commonly headache and nasopharyngitis. No meningococcal infections occurred during the trials, and one occurred following the completion of the REGAIN OLE trial that was successfully treated.

Neutralizing antibodies to the C5 monoclonal antibody or the macrocyclic peptide have not been found to a degree that inhibited the therapeutic effects of the drug.

## How Complement Inhibitors Integrate With Standard of Care Therapies

Currently, complement inhibition has been restricted by regulatory agencies in Europe and Japan for use in patients who have refractory AChR+ gMG. While the FDA did not place such a restriction for its use in the USA, the insurance industry has followed the parameters of the phase 3 REGAIN trial, limiting, for the most part, its use in a similar patient population. One hopes current trials of ravulizumab and zilucoplan will change this and allow their use in broader populations of patients and earlier in the disease course.

The accumulated data on adverse events related to complement inhibition in PNH, aHUS, and MG exceed 50,000 patient years. The adverse event profile is quite favorable when compared to adverse event profiles of current therapies, e.g., corticosteroids, purine inhibitors, cyclophilins, and other broad-spectrum immunosuppressants.

There is a single missense C5 heterozygous mutation, c.2654G → A, that predicts the polymorphism p.Arg885His in 3.5% of the Japanese population and in a Han Chinese population. This mutation prevents binding and blockade of eculizumab at the C5 domain as shown in a Japanese study of this drug in PNH (64). The REGAIN trial did not enroll patients with this mutation. Zilucoplan, a macrocyclic peptide, has a different binding site on C5 and C5b and would be effective in patients with this mutation.

A clearer understanding of the pathophysiological processes that occur at the neuromuscular junction in response to complement inhibition is needed. For instance, is there repair of the postjunctional folds with long-term use of these drugs? If such were to occur, one would have a firm argument to initiate treatment much earlier than its current use and perhaps even as primary therapy. Further study is warranted, given the rapid onset of effect, to determine the role of complement inhibition as a rescue therapy during periods of acute deterioration or in myasthenic crisis. Its role in seronegative MG and those with antibody to LRP4 is yet to be determined. While IgG1 and IgG3 antibody subclasses are reported in MuSK MG, high levels of MuSK-specific IgG1 or IgG3 have not been identified in these patients (65, 66). These data suggest complement inhibition would not be effective in IgG4-predominant mediated MG, such as MuSK MG, as this immunoglobulin subclass activates complement weakly. It is to be determined what the role of combinational therapy will play in the management of MG. As detailed above, there is no question that complement inhibition targets the primary effector mechanism for the destruction of the postjunctional folds of the neuromuscular junction. However, circulating antibody remains available to target epitopes of the AChR complex, and non-complement mechanisms driving AChR loss are not influenced. It is attractive to think of combinational treatment with an FcRn inhibitor as a means of accomplishing the task. Information regarding dosing, dosing intervals of each drug, and the predominance of one drug vs. the other will only come with further study.

The rapid, robust, and sustained improvement seen with C5 inhibition as evidenced by the clinical trials and subsequent analyses makes this treatment very favorable in patients with generalized AChR antibody-positive gMG. It has been transformational in the lives of many patients who have previously failed multiple therapies and has made significant strides in alleviating the burden of disease of chronic MG. Current trials will address its role in earlier management of the disease.

## Author Contributions

KA, HK and JH contributed to the organization of the review and each wrote sections of the manuscript. All authors contributed to manuscript revision, read and approved the submitted version.

## Conflict of Interest

HK is PI of the Rare Disease Network for Myasthenia Gravis (MGNet, NS115054) and received grant 508240 from the Muscular Dystrophy Association; is a consultant for Alnylam Pharmaceuticals, Ra Pharmaceuticals and UCB Pharmaceuticals; and is CEO and CMO of ARC Biotechnology, LLC based on US Patent 8,961,981. JH has received research support from Alexion Pharmaceuticals, Argenx BVBA, the Centers for Disease Control and Prevention (Atlanta, GA, USA), the Muscular Dystrophy Association, the National Institutes of Health (including the National Institute of Neurological Disorders and Stroke and the National Institute of Arthritis and Musculoskeletal and Skin Diseases), Ra Pharmaceuticals; honoraria/consulting fees from Alexion Pharmaceuticals, argenx BVBA. Ra Pharmaceuticals and Viela Bio, Inc. and non-financial support from Alexion Pharmaceuticals, argenx BVBA, and Ra Pharmaceuticals. The remaining author declares that the research was conducted in the absence of any commercial or financial relationships that could be construed as a potential conflict of interest. The handling editor declared a past co-authorship with several of the authors HK and JH.

## Abbreviations

Ab: Antibody
AChR: Acetylcholine receptor
aHUS: Atypical hemolytic uremic syndrome
C: Complement
DAF: Decay accelerating factor
EAMG: Experimental autoimmune myasthenia gravis
gMG: Generalized myasthenia gravis
IVIg: Intravenous immunoglobulin
IST: Immunosuppressive therapy
LRP-4: Lipoprotein receptor-related protein 4
MAC: Membrane Attack Complex
MuSK: Muscle-specific kinase
MG: Myasthenia gravis
MGC: Myasthenia Gravis Composite
MG-ADL: Myasthenia Gravis Activities of Daily Living
MGFA: Myasthenia Gravis Foundation of America
MG-QoL 15: Myasthenia Gravis Quality of Life 15
MM: Minimal manifestation
MSE: Minimal symptom expression
FcRn: Neonatal Fc receptor
OLE: Open-label extension
PTMG: Passive transfer myasthenia gravis
PNH: Paroxysmal nocturnal hemoglobinuria
QMG: Quantitative Myasthenia Gravis
sCR1: Soluble complement receptor 1
TCC: Terminal complement component.
